# Electronic health records: what does your signature signify?

**DOI:** 10.1186/1754-9493-6-20

**Published:** 2012-08-13

**Authors:** Michael S Victoroff MD

**Affiliations:** 1 Risk Management Consultant, COPIC, Inc.; Associate Clinical Professor, Dept. of Family Medicine, University of Colorado School of Medicine, 5195 E Weaver Dr, Centennial, CO, 80121-3500, USA

**Keywords:** Electronic signature, Attestation, Liability, Non-repudiation, Metadata, Electronic health records

## Abstract

Electronic health records serve multiple purposes, including clinical communication, legal documentation, financial transaction capture, research and analytics. Electronic signatures attached to entries in EHRs have different logical and legal meanings for different users. Some of these are vestiges from historic paper formats that require reconsideration. Traditionally accepted functions of signatures, such as identity verification, attestation, consent, authorization and non-repudiation can become ambiguous in the context of computer-assisted workflow processes that incorporate functions like logins, auto-fill and audit trails. This article exposes the incompatibility of expectations among typical users of electronically signed information.

## Background

Electronic media force us to examine what it means to “sign” a document. What’s the point of “signing” an entry in an Electronic Health Record? To identify authorship? Control access? Attach accountability? Prevent repudiation? All this is can be accomplished by the login information captured in metadata. Why the ceremony of a “signature?”

John Hancock knew what he was doing when he signed the Declaration of Independence. Would he be comfortable today signing documents he didn’t read, with typographical and factual errors, with content he found unsupportable or attestations of forgotten events, just to notch forward the cogwheels of commerce? When does my mark declare moral intent, versus merely showing that someone using my credentials was logged into the system? Let’s take a survey.

## The plaintiff attorney’s understanding

“Your signature on this record affirms that you have read, examined and analyzed every aspect of the attached text in exhaustive detail. You verify that every word, punctuation mark and space (including blank spaces, indentations and relative position to other items on the page) represents, in the most minute degree, precisely the thoughts, ideas and expressions which you intended at the time this document was created. You certify that the literal content is complete in every aspect, with no omissions of any kind. You have personally verified each statement, claim and assertion with external sources of unimpeachable reliability. You have also evaluated every possible implicit meaning and conceivable nuance of the attached text in full semantic detail, including imagery, puns, allusions, metaphors and usage in all modern and archaic forms of American and British English and every other language using the Latin alphabet. You hereby affirm, avow and declare that no conceivable enhancement, elision, amplification, explication or amendment could possibly improve upon the perfection of this work, which shall stand, infallibly, for all eternity, as the perfect written manifestation of your exact thoughts and actions upon the date inscribed, so help you God.”

## The defendant’s understanding

“I speculate that the markings on this document might suggest that, at some moment, I was in a position to edit the related text and was attached to a recording implement. To the best of my knowledge and belief, the record pertains to an encounter which I may have had at some previous time; or at least it evokes memories of occasions similar in some respects to what the account describes. Not having examined the content in any detail, I cannot positively say whether any specific statement might be accurate. But, that is not to be taken as an opinion whether any might be inaccurate. Essentially, I have no independent recollection of anything stated or implied; though some aspects seem vaguely familiar in a general sense. I would surmise that upon further analysis, this document might be improved by the addition of clarifying material, plus the correction of possible defects, omissions and errors which, taken together, might substantially alter the emphasis and meaning of certain statements herein – or possibly not. However, I reserve the right to revise this impression at any time.”

## The resident, hospitalist or partner’s understanding

“I am presented with notes, orders and results all day long, which I am obliged to sign or co-sign retrospectively as part of a meaningless administrative ritual. Some of these items I originated, some are the work of colleagues and many are from people I don’t know. There is no reason to read them or take the slightest note of their content, because neither judgment nor discretion enters into signing them. Most have to do with issues long past the time when my input could have any influence. Whether or not they were my own doing, I can’t retract them, even if I completely disavow what’s written. Whether my signature is needed to release an administrative barrier or satisfy a financial formality, I recognize my duty is to keep the workflow moving, and am grateful when colleagues do the same. I understand that this function is purely ceremonial and does not make me accountable for the content or subsequent effects of any item to which I affix my name.”

## The information technologist’s understanding

“We can easily replicate the contents of any field from the actively logged-in credential to populate any other location in the database. This is a bit pointless (and violates Codd’s Rules for database normalization), since the metadata log attached to each item already stamps it with the date and time to the millisecond and the GPS location of the identity used to create, change or delete any bit of data, along with the phase of the moon and the driver’s license and blood type of the purported user, while storing all prior versions of every item forever. On the other hand, we can’t tell you who actually signed in.”

## The nurse’s understanding

“We just use any available workstation. Whoever logs in first gets their name attached to everything done from that terminal all day.”

## The patient’s understanding

“I sign whatever they give me so I can get treated.”

These admittedly burlesque examples illustrate bona fide incoherence among user’s understandings about what electronic signatures imply. The problem arises largely because a single electronic document typically serves multiple purposes (e.g., a progress note might be either clinical “scratch paper” or pivotal legal testimony). This differs from paper forms, which – although any might become evidence in a legal matter – traditionally fit one purpose at a time, and their content and purpose (e.g., phone message, prescription, rounding schedule) tell users what weight to place upon the marks found on them. Ultimately, one thing technology does not change is that in disputes, all interpretations are fair game.

## Competing interest

I have reviewed the descriptions of financial and non-financial competing interests and declare that I have none.

## Author’s information

The author has over 30 years’ experience in medical informatics and bioethics. He has been an EHR developer and is a consultant in patient safety and liability risk management related to health information technology. He is Editor-in-Chief of EHREvent.org (PDR Network) and a member of the E.31 Standards Sub-Committee (EHR) of ASTM International.

